# Interactions of nebulized heparin with intravenous antithrombin for combined therapy of acute lung injury

**DOI:** 10.1186/cc9626

**Published:** 2011-03-11

**Authors:** S Rehberg, L Sousse, Y Yamamoto, LD Traber, DL Traber, P Enkhbaatar

**Affiliations:** 1 University of Muenster, Germany; 2 The University of Texas Medical Branch, Galveston, TX, USA

## Introduction

The present randomized, controlled, experimental study was performed to compare the effects of two different doses of nebulized heparin on the efficiency of the combined therapy with intravenous (i.v.) recombinant human antithrombin (rhAT) and nebulized tissue plasminogen activator (TPA) in an established ovine model of acute lung injury.

## Methods

Chronically instrumented sheep were subjected to a 40% total body surface area third-degree burn and 48 breaths of cotton smoke under deep anesthesia. Sheep were randomly assigned to receive an i.v. infusion of 6 U/kg/hour rhAT (started 1 hour post injury) combined with nebulized TPA (2 mg every 4 hour, started 4 hours post injury) and heparin (5,000 (low-dose) or 10,000 IU (high-dose), respectively, every 4 hours, started 2 hours post injury) or 0.9% NaCl i.v. and aerosolized (control; *n *= 6 each). All sheep were awake, mechanically ventilated and fluid resuscitated according to international guidelines for 48 hours. Data are expressed as mean ± SEM at 48 hours.

## Results

Both strategies attenuated lung injury, as suggested by higher PaO_2_/FiO_2 _ratios (low-dose: 276 ± 44 mmHg, high-dose: 352 ± 25 mmHg, control: 134 ± 30 mmHg) and lower airway peak pressures (27 ± 2 cmH_2_O, 27 ± 1 cmH_2_O, 36 ± 2 cmH_2_O). Notably, the combination with low-dose heparin reduced pulmonary transvascular fluid flux (16 ± 2 ml/hour, 40 ± 5 ml/hour, 51 ± 4 ml/hour) and the permeability index (9 ± 1 ml/hour, 19 ± 2 ml/hour, 25 ml/hour) and increased plasma protein (4.6 ± 0.1 g/dl, 3.9 ± 0.2 g/dl, 4.0 ± 0.3 g/dl) versus both other groups (*P *< 0.05 each). Cumulative net fluid balance was lower in the low-dose heparin group (2.1 ± 0.2l) versus control animals (3.5 ± 0.4 l; *P *< 0.05).

## Conclusions

With the lower dose of heparin the systemic anti-inflammatory effects of i.v. rhAT on vascular leakage are more pronounced, while the local, beneficial effects of nebulized heparin on gas exchange are preserved. Therefore, lower doses of heparin may be more beneficial when used in combination with i.v. rhAT for the treatment of combined burn and smoke inhalation injury. A reduction of the systemic interaction between heparin and rhAT represents a possible explanation.

